# Elicitation of Both Anti HIV-1 Env Humoral and Cellular Immunities by Replicating Vaccinia Prime Sendai Virus Boost Regimen and Boosting by CD40Lm

**DOI:** 10.1371/journal.pone.0051633

**Published:** 2012-12-07

**Authors:** Xianfeng Zhang, Tomoyoshi Sobue, Mao Isshiki, Shun-ichi Makino, Makoto Inoue, Kazunori Kato, Tatsuo Shioda, Takashi Ohashi, Hirotaka Sato, Jun Komano, Hideji Hanabusa, Hisatoshi Shida

**Affiliations:** 1 Institute for Genetic Medicine, Hokkaido University, Kita-ku, Sapporo, Japan; 2 DNAVEC Corporation, Techno Park Oho, Tsukuba, Ibaraki, Japan; 3 Department of BioMedical Engineering, Toyo University, Kawagoe, Saitama, Japan; 4 Department of Viral Infection, Research Institute for Microbial Disease, Osaka University, Yamada-oka, Suita-shi, Osaka, Japan; 5 Division of Virology, Department of Infectious Diseases, Osaka Prefectural Institute of Public Health, Nakamichi Higashinari-ku, Osaka, Japan; 6 Ogikubo Hospital, Ogikubo, Suginami, Tokyo, Japan; University of Cape Town, South Africa

## Abstract

For protection from HIV-1 infection, a vaccine should elicit both humoral and cell-mediated immune responses. A novel vaccine regimen and adjuvant that induce high levels of HIV-1 Env-specific T cell and antibody (Ab) responses was developed in this study. The prime-boost regimen that used combinations of replication-competent vaccinia LC16m8Δ (m8Δ) and Sendai virus (SeV) vectors expressing HIV-1 Env efficiently produced both Env-specific CD8^+^ T cells and anti-Env antibodies, including neutralizing antibodies (nAbs). These results sharply contrast with vaccine regimens that prime with an Env expressing plasmid and boost with the m8Δ or SeV vector that mainly elicited cellular immunities. Moreover, co-priming with combinations of m8Δs expressing Env or a membrane-bound human CD40 ligand mutant (CD40Lm) enhanced Env-specific CD8^+^ T cell production, but not anti-Env antibody production. In contrast, priming with an m8Δ that coexpresses CD40Lm and Env elicited more anti-Env Abs with higher avidity, but did not promote T cell responses. These results suggest that the m8Δ prime/SeV boost regimen in conjunction with CD40Lm expression could be used as an immunization platform for driving both potent cellular and humoral immunities against pathogens such as HIV-1.

## Introduction

An effective HIV vaccine should elicit both antibodies [1] and cell-mediated immune responses in order to control HIV infection. Since the majority of clinical isolates of human immunodeficiency virus type 1 (HIV-1) are highly resistant to neutralizing antibodies and antigenically variable, major efforts have been aimed at eliciting cellular immunity against less variable antigens. Typical prime/boost strategies using DNA and replication-defective viral vectors have been extensively examined. These regimens efficiently elicit cellular responses including cytotoxic T cells (CTL), but are less effective at eliciting humoral responses. For example, adenovirus and vaccinia virus-based vectors expressing Gag, Nef, and other components of HIV-1 have been shown, in nonhuman primates [2]–[5] and in human trials [6], [7], to elicit considerable multifunctional T cell responses and control early viral replication to some extent. These preparations, however, did not induce a sufficient level of immunity to protect vaccinees from HIV/simian immunodeficiency virus (SIV) infection in the absence of neutralizing antibodies [8]. Therefore, more potent immunogens and better vaccination regimens are required.

The RV144 trial that included priming with a recombinant canarypox vector, ALVAC-HIV vCP1521, followed by booster with the HIV-1 envelope gp120 protein, AIDSVAX gp120 clades B and E, plus an alum adjuvant showed a modest level of efficacy in reducing HIV-1 infection rates in Thailand [9]. Extended analysis of this HIV vaccine trial showed that it is the vaccine trial to succeed in eliciting IgG antibodies to the V1V2 region of Env, and the presence of these antibodies were inversely correlated to the rate of infection [10], suggesting an importance to elicit anti HIV-1 specific antibodies. Accordingly, both antibodies and cell-mediated immune responses should be considered for the vaccine development in order to control HIV infection.

Replication-competent vaccinia virus (VV) that has been proven to be safe in human vaccination against smallpox may be a good vehicle candidate. Among several vaccinia strains, LC16m8 has an extremely low neurovirulence profile, comparable to the replication incompetent vaccinia viruses MVA and NYVAC, and is safe in immune compromised animals [11]–[13]. LC16m8 is able to induce immunity at levels similar to the original Lister (LO) strain and the US licensed vaccine dryvax strain [11]–[13], and no serious adverse effects were detected in the administration of LC16m8 to 100,000 infants and 3,000 adults [14]. However, LC16m8 is genetically unstable and can spontaneously generate more virulent revertants. To improve the safety of LC16m8, we identified the B5R gene responsible for the reversions and constructed the genetically stable LC16m8Δ (m8Δ), which is essentially the same as LC16m8 in antigenicity, safe in mice and rabbits, and much more immunogenic than the MVA strain [13]. Thus, m8Δ may be a better vehicle for vaccines. Indeed, immunization in a prime-boost strategy using DNA and m8Δ expressing SIV Gag elicited 7–30 fold more IFN-γ producing T cells in mice than were produced using the non-replicating vaccinia DIs strain [15].

**Figure 1:**
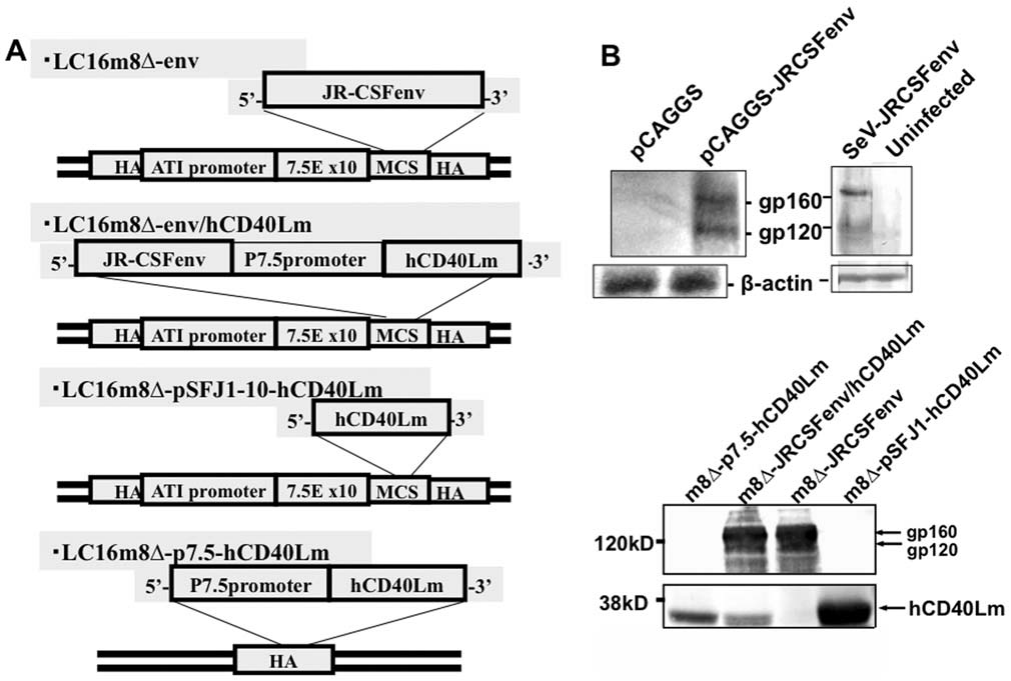
Structures and expression of recombinant DNA and LC16m8 Δ. (A) Structures of four different recombinant m8Δs. (B) Profiles of Western blotting for Env and human CD40Lm expressed by infection with the HIV-1 Env expression plasmid, pCAGGS-JRCSFrev/env, SeV-env, and recombinant m8Δs are presented.

The Sendai virus (SeV) is a non-segmented negative-strand RNA virus belonging to the paramyxovirus family and is considered nonpathogenic in humans [16]–[19]. A SeV vector expressing the SIV gag gene elicits SIV-specific CTL very efficiently and controlled SIV replication in a subset of immunized macaques [20], [21]. Thus, the SeV vector may be another candidate for a better immunogen.

**Figure 2:**
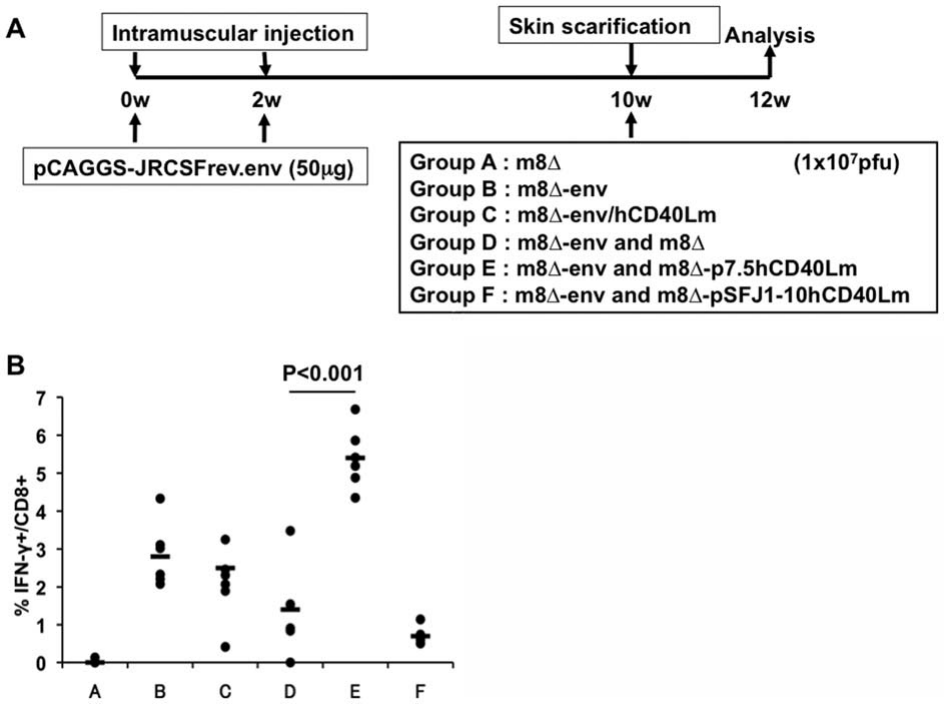
Cellular immunity elicited by a regimen consisting of DNA priming followed by vaccinia m8 Δ **boosts.** (A) Schematic schedules of DNA prime/m8Δ-Env boost vaccination protocol. Mice were immunized twice with pCAGGS-JRCSFrev/env and boosted with various vaccinia viruses as follows. Group A: m8Δ; group B: m8Δ-Env; group C: m8Δ-Env/hCD40Lm; group D: m8Δ-Env plus m8Δ; group E: m8Δ-Env plus m8Δ-p7.5hCD40Lm; and group F: m8Δ-Env plus m8Δ-pSFJ1-10hCD40Lm. (B) Comparison of Env peptide-specific CD8^+^ T cell responses. The frequencies of IFN-γ^+^ CD8^+^ T cells in gated CD8^+^ T cell compartment was determined by intracellular cytokine staining (ICS) and FACScalibur/FACScanto analysis.

**Table 1:** Mice immunization protocol used in this study.

Vaccination group	Prime	Boost
A	pCAGGS-JRCSF rev.env	M8Δ
B	pCAGGS-JRCSF rev.env	M8Δ-env
C	pCAGGS-JRCSF rev.env	M8Δ-env/hCD40Lm
D	pCAGGS-JRCSF rev.env	M8Δ-env+ m8Δ
E	pCAGGS-JRCSF rev.env	M8Δ-env + m8Δ-p7.5hCD40Lm
F	pCAGGS-JRCSF rev.env	M8Δ-env + m8Δ-pSFJ1-10hCD40Lm
G	M8Δ-env + m8Δ-p7.5hCD40Lm	SeV-JRCSF env
H	M8Δ-env + m8Δ	SeV-JRCSF env
I	M8Δ-env/hCD40Lm	SeV-JRCSF env
J	M8Δ-env	SeV-JRCSF env
K	M8Δ	SeV-JRCSF env
L	Naive	SeV-JRCSF env
M	pCAGGS-JRCSF rev.env	SeV-JRCSF env

In addition to adopting better vaccination vehicles, combining these with an immune stimulating factor could produce a better efficacy. The CD40 ligand (CD40L, CD154), which belongs to the tumor necrosis factor (TNF) family, is a 39 kDa type II membrane glycoprotein that is predominantly expressed on activated CD4^+^ T cells [22]. CD40, the TNF receptor superfamily member that is the CD40L receptor, is expressed on all antigen-presenting cells (APCs), including macrophages, dendritic cells (DCs) and B lymphocytes [23]. Interactions between these receptors and ligand play a central role in adaptive immune responses including maturation of DCs and class switching of immunoglobulin genes [24]. Coexpression of CD40L with immunogens has the potential to enhance both humoral and cellular immune responses in various regimens [25]–[29]. However, one concern is that high levels of CD40L, mainly resulting from cleavage to produce a soluble form, may have deleterious side effects and could lead to systemic inflammatory responses and cardiovascular disease. A non-cleavable CD40L, CD40Lm, which was constructed with point mutations in the membrane proximal region, was reported to be less toxic in vivo [30]. Therefore, coexpression of CD40Lm may further enhance the induction of immune responses to HIV-1 without adverse effect.

**Figure 3:**
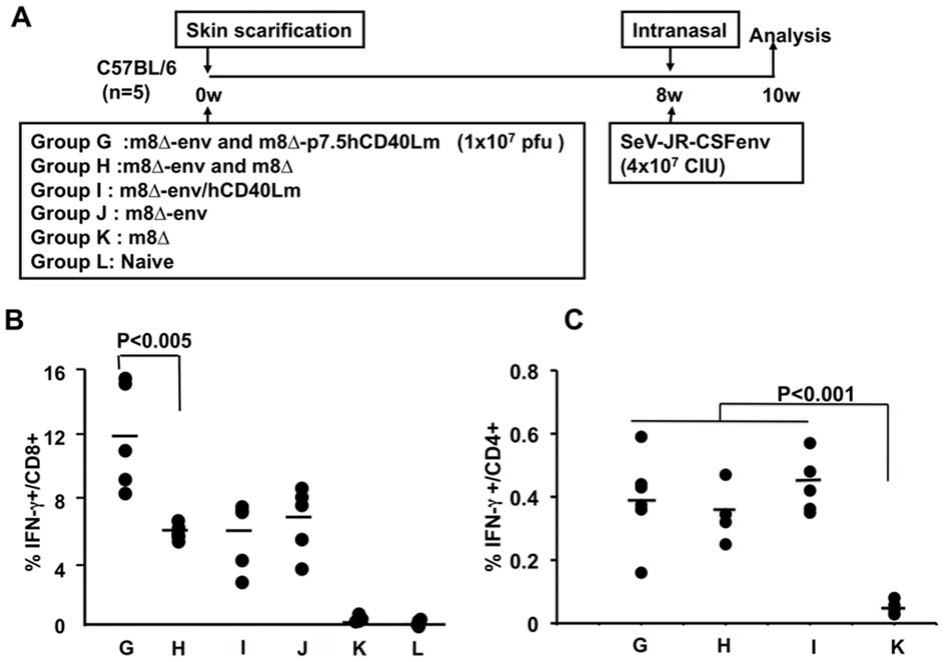
Immunity elicited by Env and CD40Lm expressing m8 Δ prime followed by recombinant SeV boost. (A) Schematic schedules of m8Δ prime/rSeV-Env boost vaccination protocol. Mice were primed with various vaccinia viruses followed by Env expressing SeV boost. Group G: primed with m8Δ-Env plus m8Δ-p7.5hCD40Lm; group H: m8Δ-Env plus m8Δ; group I: m8Δ-Env/hCD40Lm; group J: m8Δ-Env; group K: m8Δ; group L: naive. (B) Comparison of Env peptide-specific CD8^+^ T cell responses. The percentage of IFN-γ^+^ expressing cells in gated CD8^+^ T cell compartment was measured by ICS and FACS analysis. (C) The percentage of IFN-γ^+^ T cells in gated CD4^+^ T cell compartment.

To identify an improved vaccination regimen that elicits higher levels of anti-HIV-1 humoral and cellular responses, various combinations of vaccine preparations were tested in this study using the vaccinia virus m8Δ and SeV vectors expressing HIV-1 Env in conjunction with the coexpression of human CD40Lm (hCD40Lm).

**Figure 4:**
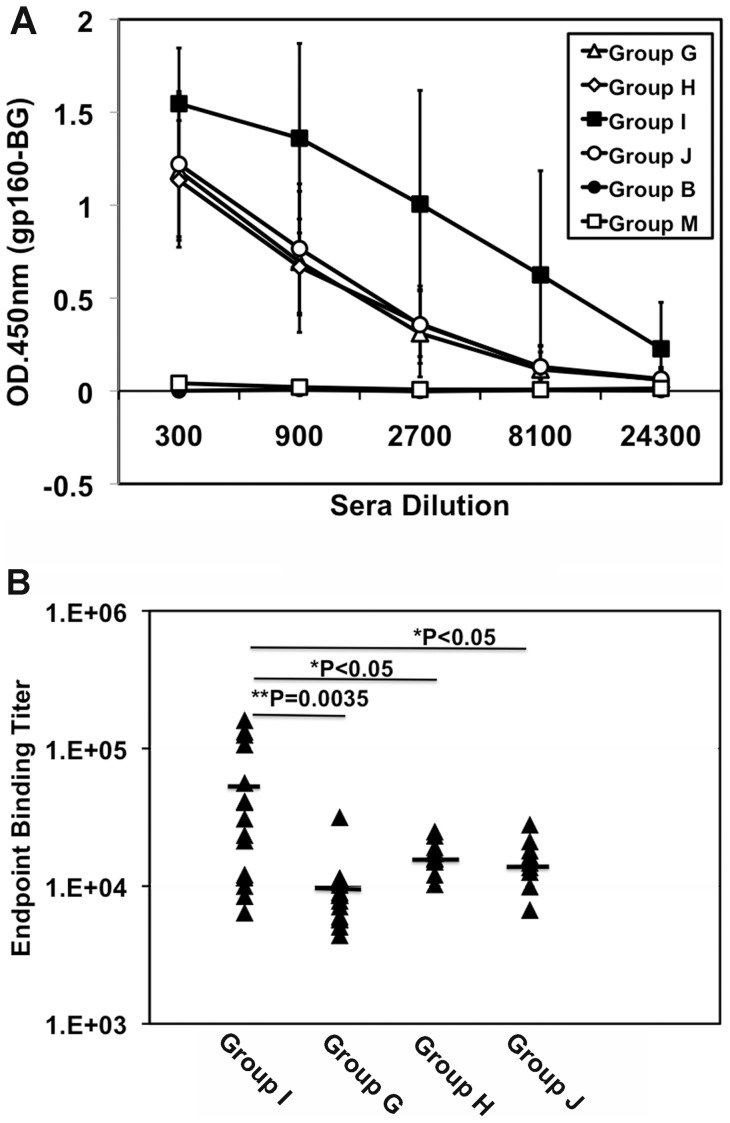
Priming with the coexpression vector m8 Δ-Env/hCD40Lm induces greater amounts of Env-specific antibodies. Serum from individual immunized mice was analyzed using an HIV-1_JR-CSF_ gp160 ELISA assay. The plates were developed with HRP-conjugated anti-mouse IgG antibody. (A) The titer of Env-specific antibodies was determined by OD_450_ values subtracted from the background values. Data are mean ± SD of the Env-specific antibody titer of all animals in each group (n = 16 for group G and I; n = 8 for group H and J; n = 5 for group B and M). (B) Endpoint binding titer of sera against HIV-1 JR-CSF gp160 from each of vaccine groups was plotted. The titer in the group primed with m8ΔEnv/hCD40Lm was significantly different from all other groups. There are no significant differences in env-specific antibody titer among the group G, H and J.

## Materials and Methods

### Ethics Statement

All animal experiments were conducted according to the *Guide for the Care and Use of Laboratory Animals*, Institute for Genetic Medicine, Hokkaido University. Study approval was issued by the Animal Care Committees of Hokkaido University.

**Figure 5:**
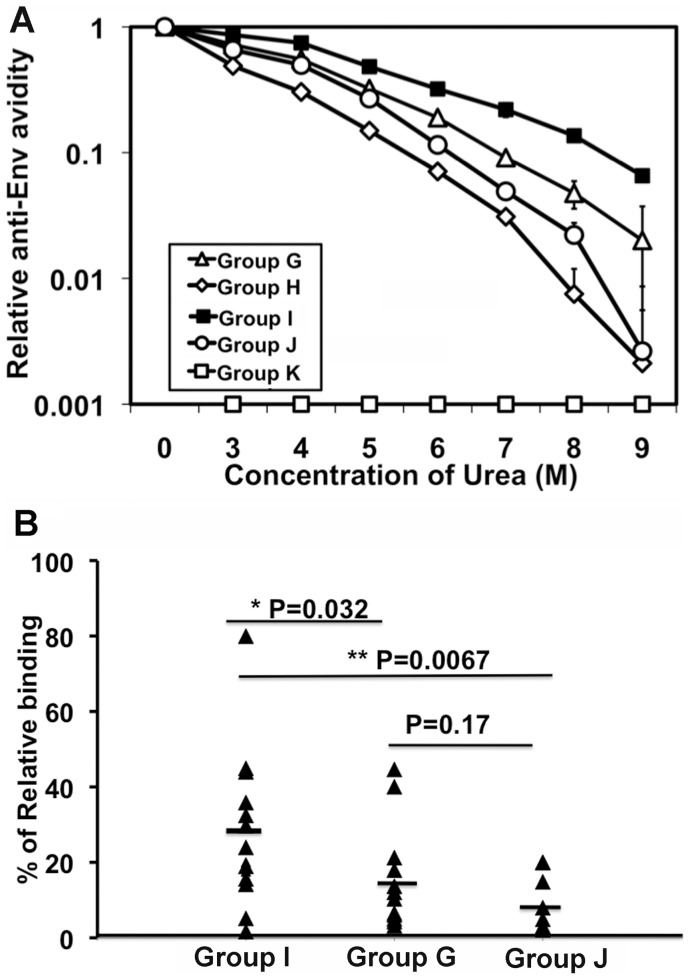
Priming with m8 Δ-Env/hCD40Lm elicits Env-specific antibodies with higher avidity. The avidity of serum antibodies for HIV-1 Env was determined based on the resistance of antibody-gp160 binding to disruption by treatment with urea. (A) Pooled serum from immunized mice of each group was applied to a HIV-1_JR-CSF_ gp160-coated ELISA plate at a 1∶300 dilution, as described above. After antibody-antigen binding, the plates were treated with increasing concentrations of urea (3–9 M) for 30 min at room temperature, followed by the ELISA procedure. (B) Dissociation analysis of 300 fold-diluted serum from individual mice using 7 M urea. The percentage of antibody remaining after urea treatment was plotted, and the mean and P values were calculated.

### Construction of HIV-1 envelope expression plasmids

The region encoding the Rev and Env genes of the HIV-1 _JR-CSF_ genome (5981–8782 nt) was inserted into the EcoR1 restriction site of the mammalian expression vector pCAGGS [31], [32] to generate pCAGGS-JRCSFrev/env. To confirm the expression of gp160, a sequence-verified pCAGGS-JRCSFrev/env was transfected into 293T cells using polyethylenimine (Polysciences, Warrington, US) [33]. Forty-eight hours after transfection, 293T cell lysates were collected and proteins were fractioned on 10% SDS polyacrylamide gels and transferred to a nitrocellulose filter (Schleicher & Schuell). Immunoblot analysis was performed with HIV-1-infected human antiserum and alkaline phosphatase-conjugated anti-human IgG (Promega, Sunnyvale, US), and then visualized using NBT/BCIP.

**Figure 6:**
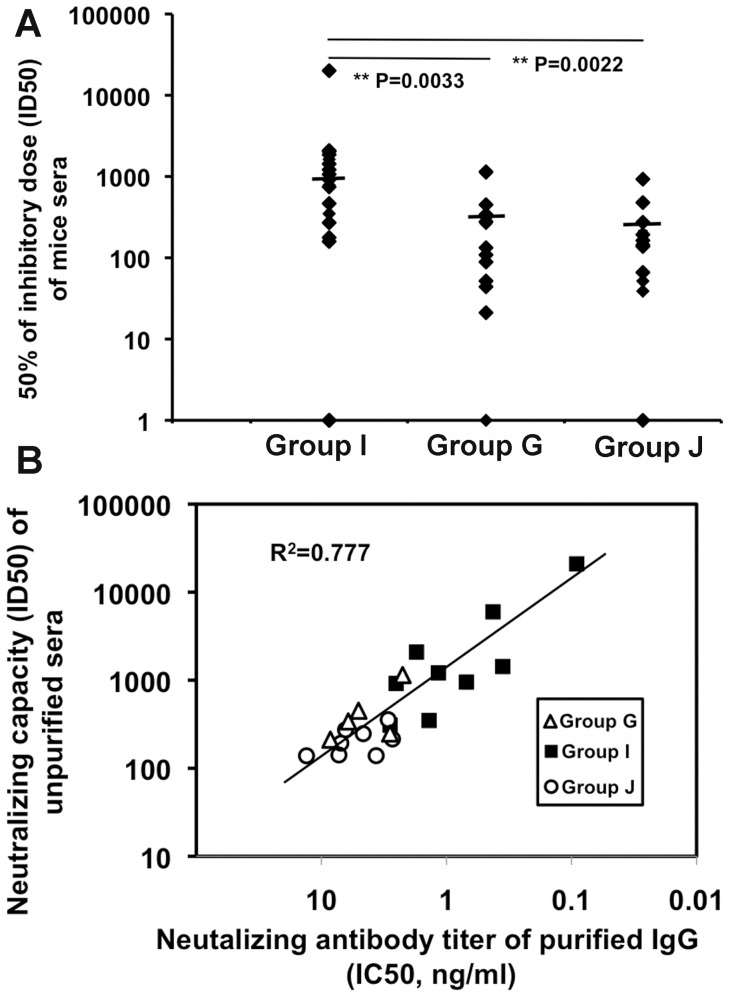
Neutralizing antibodies elicited in the Env expressing LC16m8 Δ prime/SeV boost regimen. (A) ID_50_ of individual mouse serum against SF162 envelope-pseudotyped virus was determined using TZM-bl cells. Groups I, G and J consisted of 21, 17 and 14 mice, respectively. (B) Antibodies from randomly selected mice sera from group G, I, J and control were purified using protein A Sepharose. ID_50_ of individual mouse serum against SF162 envelope-pseudotyped virus vs 50% inhibitory concentration (IC_50_) (concentration of purified IgG that caused a 50% reduction in the RLU compared to virus control) of individual purified IgG was plotted. The regression line and R square are shown.

### Construction of recombinant LC16m8Δ

The four strains of recombinant m8Δ used in this study are constructed as follows. To construct a m8Δ that expresses the full HIV-1 _JR-CSF_ Env gene under the control of the vaccinia virus promoter in pSFJ1-10 [21], the AvrII-XhoI fragment of the JR-CSF genome was subcloned into the pJW322 plasmid [34] that had been digested with AvrII and SalI, and the vaccinia virus transcription termination signals (TTTTTNT) in the envelope gene were synonymously mutated using an in vitro mutagenesis kit (Stratagene). The coding region for gp160 was then amplified by PCR using the forward primer TTTCGGACCGCCACCATGAGAGTGAAGGGGATCAGG (underline shows the RsrII site) and the reverse primer ATAGGCCGGCCTTATAGCAAAGCCCTTTCCAAGC (underline shows the FseI site). The PCR product was ligated into the LC16m8Δvnc110 [15] genome that had been digested with FseI and RsrII. The ligated DNA was transfected into BHK cells that were infected with canarypox virus, as described previously [14]. The vaccinia virus constructed was designated LC16m8Δ-env. For the m8Δ-p7.5hCD40Lm construct, the human CD40Lm gene [35] was inserted into pVR1 containing the p7.5 vaccinia virus promoter [36] at the BamHI and AvaI sites, which interrupts the HA gene sequence [37], [38]. The construct was transfected into m8Δ-infected BHK cells, followed by selection based on an HA^−^ phenotype, as described previously [38], [39]. For the m8Δ-pSFJ1-10hCD40Lm construct, the hCD40Lm gene was inserted into the XmaI and Not I sites of pBHAR that contains the pSFJ1-10 sequence inserted within the vaccinia virus HA gene [40]. For the m8Δ-Env/hCD40Lm construct that coexpresses pSFJ1-10-driven Env and p7.5-driven hCD40Lm, the Env gene fragment with EcoRI and SacI sites, and the p7.5 promoter-hCD40Lm fragment with SacI and XmaI sites were generated by PCR and the construct was inserted into the EcoRI and XmaI sites of pBR322. Then, the entire env-p7.5 promoter-hCD40Lm region was amplified by PCR and ligated into the m8Δvnc110 genome, as above. A schematic diagram of each m8Δ construct is shown in Fig. 1A. To verify protein expression, RK13 cells were infected with these recombinant m8Δs at a moi of 5 and cultured for 24 h at 33°C. Cell lysates were prepared, and analyzed by immunoblot analysis using the HIV-1-infected human antiserum or mouse anti-hCD40Lm mAb as the primary antibodies.

**Figure 7:**
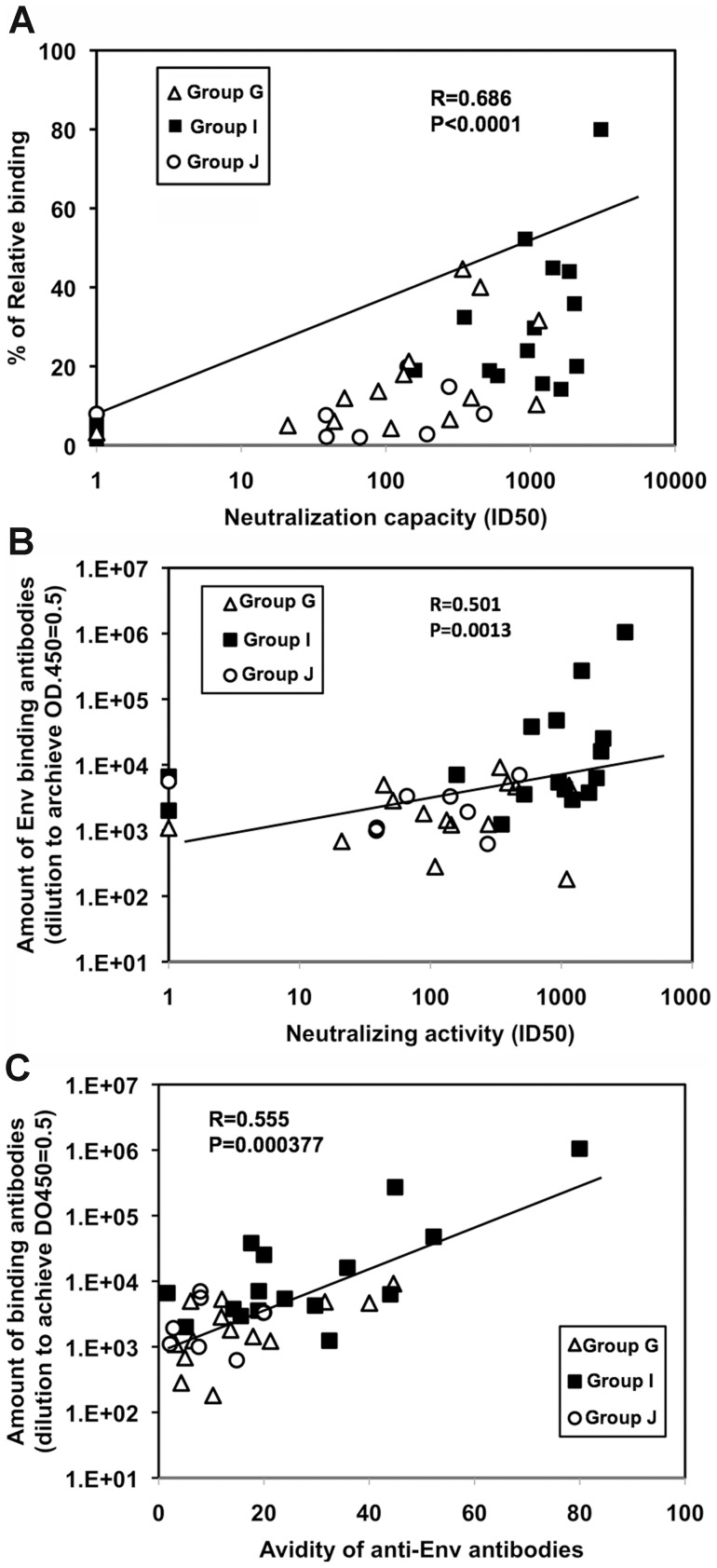
Correlation between neutralization activity against HIV-1 SF162 and the amount and avidity of anti-Env antibodies. The trendline and R and P values are displayed. (A) Correlation between neutralization activity against HIV-1 SF162 and the avidity of Env antibodies. (B) Correlation between the amount of anti-Env antibodies and neutralization activity. (C) Correlation between the amount of anti-Env antibodies and their avidity.

### Construction of SeV expressing the JR-CSF env gene

Potential EIS sequences that may affect transcription of SeV were identified in the JR-CSF Env gene, and nucleotide substitutions that did not alter the amino acid sequence were made using in vitro mutagenesis with the following primers: mutation 1 forward primer, CCATCGTCTTCACTCACTCCTCAGGAGGGGATCCAGAAATTG; mutation 1 reverse primer, GAATAACACTTT AAAACAGATAGTTGAGAAGCTCCGCGAGCAGTTCAACAACAAGACCATCGTCTTCACTCACTCCTCAGGAG; mutation 2 forward primer, GTGAAGATCGAACCATTAGGAGTAGCACCCACCAAGGCAAAG; mutation 2 reverse primer, GAGACATGAGGGACAATTGGAGAAGTGAGCTCTACAAGTACAAGGTCGTGAAGATCGAACCATTAGGAGTA; mutation 3 forward primer, CGCATCGTG TTCTCTGTACTTTCTATAGTGAATAGAGTTAGGCAGG; and mutation 3 reverse primer, GTTTGACATAACAAAATGGCTGTGGTACATCAAGATCTTCATCATGA TCGTGGGAGGCCTGATCGGTCTCCGCATCGTGTTCTCTGTACTTTCTATAG. The mutated env fragment was subcloned into pSeV/ΔF and replication incompetent SeVJRCSFenv recombinant virus was constructed as previously described [41].

**Figure 8:**
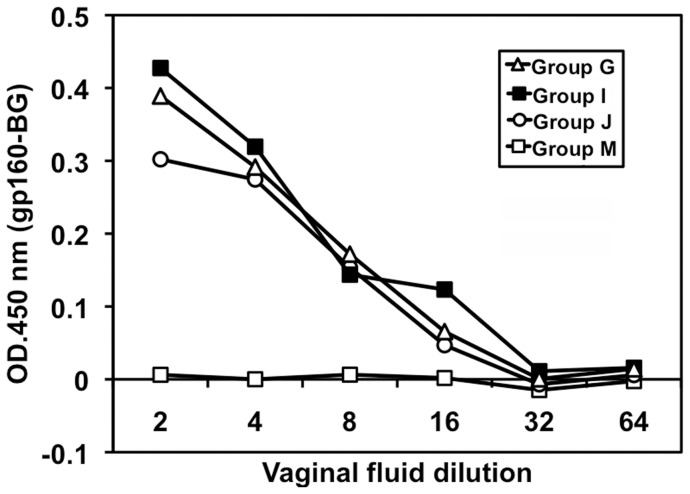
Env-specific mucosal IgG elicited by the regimen of Env expressing LC16m8 Δ prime/SeV boost. Vaginal fluid pooled from 5–6 immunized mice was analyzed by HIV-1_JR-CSF_ gp160 ELISA assay. The ELISA was performed as indicated in Fig. 4. The data shown are representative results of three independent experiments.

### Immunization of mice

Seven-week-old female C57BL/6J mice (CLEA Japan, Tokyo) were primed twice at 2 week intervals by intramuscular (i.m.) injection with 100 µl PBS containing 50 µg pCAGGS-JRCSFrev/env. Eight weeks later, animals were boosted with 1×10^7^ PFU of the recombinant m8Δ by skin scarification (s.s.) [42]. Alternatively, mice were primed with 1×10^7^ PFU of the recombinant m8Δ by s.s. and eight weeks later boosted with 4×10^7^ CIU of recombinant SeV intranasally (i.n.). Two weeks after the last immunization, vaginal fluids were collected, and mice were then sacrificed to collect sera and spleens.

### Intracellular cytokine staining (ICS)

Splenocytes were stimulated with 10 µg/ml HIV-1 consensus subtype B Env (15-mer) peptides (AIDS Research and Reference Reagent Program) in the presence of Alexa Fluor-488 labeled anti-mouse CD107a (2.5 µg/ml) and brefeldin A (BD Biosciences) for 6 h. The cells were then washed and stained with PE-labeled anti-mouse CD8 (eBioscience, San Diego, US) and Pacific Blue-labeled anti-CD4 (eBiosience, San Diego, US) mAbs for 30 min at 4°C. After washing, the cells were permeabilized with Cytofix/Cytoperm solution (BD Bioscience, Franklin lakes, US) and stained with APC-labeled anti-mouse IFN-γ (eBiosience, San Diego, US) mAb. Then, the samples were subjected to analysis using a FACScalibur or FACScantoII instrument (BD Bioscience, Franklin lakes, US). Data were analyzed with the FlowJo software (Tree Star). The frequencies of IFN-γ+ or IFN-γ, CD107a double positive T cells among CD4 or CD8 gated lymphocytes were determined.

### Purification of IgG from mouse serum

Twenty micro liter of mice sera randomly selected from group G, I, J and control were mixed with 20 µl of protein A Sepharose 4 Fast Flow resins (GE healthcare life science, Tokyo, Japan) that was pre-washed with TBS buffer (10 mM Tris-HCl, pH 7.5, 150 mM NaCl), and incubate at 4°C for 1 hr with rotation. The Protein A sehparose resins were then rinsed 3 times with TBS buffer. The antibodies were eluted with 50 µl of elution buffer (50 mM glycine-HCl, pH 2.5) at 4°C for 5 min followed by addition of 2.5 µl of 1 M Tris-HCl (pH 9.0) to adjust pH to 7. The isotype of the purified antibodies were analyzed with BD cytometric bead array system according to the manufacturer's instruction.

### Serum antibody measurements by enzyme-linked immunosorbent assay (ELISA)

To prepare ELISA plates coated with HIV-1_JR-CSF_ gp160, 293T cells (2.5×10^6^ cells) that had been transfected with 10 µg of pCAGGS-JRCSFrev/env 2 days before were lysed in 1 ml of TMN buffer (10 mM Tris-HCl, pH 7.4, 1.5 mM MgCl_2_, 140 mM NaCl, 0.5% NP40 and 1X complete protease inhibitor cocktail (Roche Applied Science, Sandhofer Strasse, Germany)) and desalted by ultrafiltration using a Centricon YM-100 (Millipore). After dilution to a final concentration of 3.0 µg/ml protein with coating buffer (eBioscience, San Diego, US), the cell lysates were added to Maxisoap 96 well ELISA plates (NUNC) at 100 µl aliquots/well and incubated overnight at 4°C. The wells were washed twice with PBS plus 0.05% Tween 20 (PBS-T) and blocked with PBS-T containing 5% skim milk for 1 h at room temperature. Subsequently, 100 µl samples of sera, serially diluted with the blocking solution, were added to the wells and incubated for 2 h at room temperature. Plates were washed five times with PBS-T. Aliquots (100 µl) of horseradish peroxidase (HRP)-conjugated anti-mouse IgG (Promega, Sunnyvale, US), diluted 1∶2500 from the stock solution, were added and incubated for 1 h at room temperature. The plates were washed five times with PBS-T and TMB ELISA substrate solution (eBioscience, San Diego, US) was added. After a 15 min incubation at room temperature, the reaction was stopped by addition of 1 M H_3_PO_4_. Optical density at 450 nm (OD_450_) was measured using a plate reader (PerkinElmer). To calculate the amounts of Env-specific antibodies, OD values were background subtracted using the OD_450_ for wells coated with cell lysates prepared from pCAGGS-transfected 293T cells.

To assess the avidity of anti-Env antibodies, 300 fold-diluted sera were added to the wells of ELISA plates and incubated as above. The plates were then washed with 3–9 M urea and subjected to the above procedure for quantification of anti-Env antibodies. Relative avidity was estimated as the ratio of absorbance after and before the urea wash. The concentration of urea required to release 50% of the bound antibody (half-maximal effective dose (ED_50_)) was calculated by linear regression analysis of plots of the probit values vs. urea concentration.

### Neutralizing antibody measurements

The titer of neutralizing Abs (nAbs) was assessed based on the reduction of luciferase reporter gene expression in TZM-bl cells after a single round of Env-pseudotyped virus infection, as described previously [35], [43]. HIV-1 Env-pseudotyped virus (1000 TCID50/ml) was incubated with five-fold serially-diluted test sera in triplicate. One set of control wells received the cells plus virus (virus control), another set of wells received cells only (background control). To examine the non-specific effect of sera, MuLV Env or VSV glycoprotein-coated pseudotype virus was included. The 50% inhibitory dose (ID_50_) was calculated as the serum dilution that caused a 50% reduction in the relative light unit (RLU) values compared to virus control wells after subtraction of background RLU.

### Measurement of IgA and IgG in vaginal fluid

To collect vaginal samples, a total of 400 µl of PBS was flushed into the vagina and collected into a microcentrifuge tube. The mucosal washings were centrifuged at 1000 g for 10 min. The supernatants were collected and stored at −20°C until assay. Total IgA or IgG concentration in the vaginal fluid was quantified using a mouse IgA/IgG ELISA quantitation kit (Bethy Laboratories, Montgomery, US) according to the manufacturer's instructions. The amount of HIV-1 gp160-specific IgA or IgG was determined using ELISA plates prepared as described above. Aliquots (100 µl) of two-fold serially-diluted vaginal samples were used. HRP-conjugated anti-mouse IgA (Bethyl Laboratories; 1∶10,000) or HRP-conjugated anti-mouse IgG (Promega, Sunnyvale, US) was used as the secondary antibody at a dilution of 1∶2500.

### Statistical analysis

Data were expressed as arithmetic mean ± standard deviation (mean ± SD). Data analysis was conducted using Student's *t*-test (EXCEL version 11.5, Microsoft). A *P*-value of <0.05 was considered significant.

## Results

### *In vitro* Expression of Env and hCD40Lm

We first confirmed the expression of the Env and hCD40Lm genes by the vectors constructed in this study by immunoblot assay. As shown in Fig. 1B, 293T cells transfected or infected with the recombinant Env expression plasmid pCAGGS-JRCSFrev/Env, or the Sendai virus (SeV) vector expressing HIV-1_JR-CSF_ envelope gene, produced gp120 and gp160. Four recombinant m8Δs were examined: m8Δ-Env carrying only the HIV-1_JRCSF_ envelope protein; m8Δ-Env/hCD40Lm carrying the envelope protein and hCD40Lm; and m8Δ-pSFJ1-10-hCD40Lm and m8Δ-p7.5-hCD40Lm, carrying hCD40Lm under different promoters, pSFJ1-10 and p7.5, respectively (Fig. 1B). Cells infected with the Env gene harboring m8Δs expressed mainly gp160 and lesser amounts of gp120 (Fig. 1B). To determine whether functional envelope proteins were produced, the m8Δ recombinants were used to infect TZM-bl cells that express CCR5 and CD4 and examined whether cell fusion could be provoked. We observed large fused TZM-bl cells, but HeLa cells infected with these viruses did not fuse (data not shown). These data indicate that functional gp120 and gp41 were produced. In cells infected with hCD40Lm harboring m8Δs, authentic hCD40Lm protein was produced and cells infected with m8Δ-pSFJ1-10-hCD40Lm expressed more CD40Lm than those infected with m8Δ-p7.5-hCD40Lm or m8Δ-Env/hCD40Lm (Fig. 1B). These data are consistent with the relative strong potency of the pSFJ1-10 promoter vs. the p7.5 promoter [40].

### Effect of hCD40Lm in the Env expressing DNA prime/LC16m8Δ boost regime

The effect of CD40Lm in improving the immunogenicity of the vaccination regime was first examined using the most frequently tested regime of priming with an Env-expressing plasmid followed by boosting with Env-expressing vaccinia m8Δ (Table 1 group A–F). Female C57BL/6J mice were primed with 50 µg of pCAGGS-JRCSFrev/env followed by boosting with 1×10^7^ PFU of various m8Δ recombinants: m8Δ (group A), m8Δ-Env (group B), m8Δ-Env/hCD40Lm (group C), m8Δ-Env and m8Δ (group D), m8Δ-Env and m8Δ-p7.5hCD40Lm (group E), and m8Δ-Env and m8Δ-pSFJ1-10hCD40Lm (group F) (Fig. 2A). Mice were sacrificed two weeks after the final immunization, and splenocytes and sera were collected for immunological assays. An ICS FACS analysis was conducted to identify HIV-1 Env-specific IFN-γ secreting CD8^+^ T cells after stimulation with a consensus subtype B Env (15-mer) peptide pool. Initially, all overlapping Env peptides representing entire JR-CSF Env were tested for their ability to induce most effective response (frequency of IFN-γ secreting CD8^+^ T cells), then the two most immunogenic peptides, 805–819 aa and 809–823 aa, were selected and the mixture of these two peptides was used thereafter. Co-immunization of m8Δ-Env with the lower expression hCD40Lm vector, m8Δ-p7.5hCD40Lm (group E), significantly enhanced the number of IFN-γ secreting CD8^+^ T cells by approximately 2–4 fold compared to the groups boosted with m8Δ-JRCSFenv alone (group B) or m8Δ-JRCSFenv together with empty m8Δ (group D). In contrast, the higher expression hCD40Lm vector, m8Δ-pFSJ1-10hCD40Lm (group F), decreased the number of IFN-γ expressing CD8^+^ T cells. There was no significant difference in the number of IFN-γ secreting CD8^+^ T cells between mice boosted with the coexpression vector, m8Δ-Env/hCD40Lm (group C), and mice boosted with m8Δ-Env (group B) (Fig. 2B). IFN-γ and CD107a double positive CD8^+^ T cell fractions were also quantified to determine the number of functional CD8^+^ T cells. As in the case of IFN-γ secreting CD8^+^ T cells, co-immunization with m8Δ-p7.5hCD40Lm enhanced the immunogenicity of m8Δ-env (data not shown). Interestingly, the expression of an optimal amount of hCD40Lm was important for the enhancement since high levels of expression were not effective (Compare group E and F in Fig. 2B). The m8Δ-pFSJ1-10hCD40Lm vector (group F) was therefore omitted from subsequent experiments. The data above suggest that uncleaved CD40L delivered by m8Δ vector help the Env expression pox vector to generate more effective T cell responses, but only when CD40Lm are expressed by the separate virus.

Although we tried to detect HIV-1_JR-CSF_ Env-specific antibodies by ELISA and TZM-bl cell-based assays, the DNA prime/m8Δ boost immunization regimens did not elicit any anti-Env antibodies (see Fig. 4, group B as representative data).

### Cellular immunity elicited in the Env expressing LC16m8Δ prime/SeV boost regimen

To identify vaccination methods that may elicit both cellular and humoral immunity, a novel immunization regimen using SeV and m8Δ expressing HIV-1_JR-CSF_ Env was tested. At first we performed a preliminary experiment to optimize the order of prime-boost regime, in which, an m8Δ-Env prime/SeV-env boost regime produced better responses than a SeV-env prime/m8Δ-Env boost regime (data not shown). The priming effects of various combinations of m8Δ recombinants such as m8Δ-Env plus m8Δ-p7.5hCD40Lm (group G), m8Δ-Env plus m8Δ (group H), m8Δ-Env/hCD40Lm (group I), m8Δ-Env (group J), and m8Δ (group K) were examined; untreated mice were the control (group L) (Fig. 3A). A higher frequency of IFN-γ^+^ CD8^+^ T cells was detected in the m8Δ-Env prime/SeV-env boost regime than in the DNA prime/m8Δ-Env boost regimen (compare Fig. 2B and Fig. 3B). Immunization with the coexpression vector m8Δ-Env/hCD40Lm (group I) did not enhance the number of IFN-γ^+^ CD8^+^ T cells compared to m8Δ-Env alone (group J), similar to the DNA prime/m8Δ boost (see Fig. 2). In contrast, the group G, in which mice were primed with a combination of m8Δ-Env and m8Δ-p7.5hCD40Lm, significantly increased the frequency of Env-specific IFN-γ secreting CD8^+^ T cells compared to the group that was primed with m8Δ-Env and empty m8Δ (group H, p<0.005) (Fig. 3B). A more detailed analysis of IFN-γ^+^ CD107a^+^ CD8+ T cells by comparison of groups G, H, I and K showed similar results in that m8Δ-p7.5hCD40Lm co-immunization with m8Δ-Env increased the number of functional CD8^+^ T cells (data not shown). These results indicate that the m8Δ prime and SeV boost regime efficiently elicits Env-specific cellular immunity, and that the inclusion of hCD40Lm delivered by a separate vector enhances cellular immunity elicited by m8Δ-Env. We also compared the frequency of IFN-γ secreting cells in CD4^+^ T cell populations (Fig. 3C). The CD4^+^ T cells responses to Env peptides 805–819 aa and 809–823 aa, stimulation were not enhanced by hCD40Lm expressed by either the same or separate vector (compare group G, H and I). Although the frequencies of IFN-γ^+^ CD4^+^ T cells were much lower than those of CD8^+^ T cells, all were significantly higher than empty m8Δ (group K). The relatively lower CD4+ T cell response might be due to the poor match of the peptides to the MHC-II molecules. Therefore, we performed epitope mapping using overlapping Env peptides that cover the entire Env protein, but no peptide induced more efficient CD4^+^ T cell response than the two peptides used above (data not shown), ruling out the possibility that stimulation with Env peptides 805–819 aa and 809–823 aa did not faithfully reflect authentic CD4^+^ T cell response.

### Antibodies elicited in the Env expressing LC16m8Δ prime/SeV boost regimen

To determine whether anti-Env antibodies were elicited, ELISAs were conducted using gp160-coated plates. All regimens involving vaccinia virus prime/SeV boost efficiently elicited anti-Env antibodies (Fig. 4, group G–J), indicating that our novel immunization regimen of m8Δ prime/SeV boost is able to elicit both humoral and cellular immune responses. In contrast, the conventional immunization regimen of DNA prime/vaccinia virus boost elicited only cellular immunities, not antibody responses. Although priming with the coexpression vector m8Δ-Env/hCD40Lm (group I) did not enhance Env-specific cellular immunity, it did elicit significantly more anti-Env antibodies than m8Δ-Env alone. Co-immunization with m8Δ-Env and m8Δ-p7.5hCD40Lm (group G) did not significantly augment the anti-Env antibody production compared to priming with m8Δ-Env alone or m8Δ-Env plus m8Δ (group J and H). Comparison of the endpoint dilution titer of the antibodies, suggests that m8Δ-Env/hCD40Lm priming (group I) elicited approximately 5–6 times more anti-Env antibody than the other immunization groups (Fig. 4B).

Next, the avidity of the anti-Env antibodies was assessed based on the relative amount of antibody that remained on the ELISA plate after a urea wash (Fig. 5). The ED_50_ of the urea wash for the anti-Env antibodies prepared from mice primed with m8ΔEnv/hCD40Lm, m8ΔEnv + m8ΔhCD40Lm, m8ΔEnv and m8ΔEnv+m8Δ (group I, G, J and H) is 5.16 M, 4.22 M, 3.84 M and 2.57 M, respectively (Fig. 5A). The relative amount of residual anti-Env antibody from group I was significantly higher than any other group after washing with 7 M urea (Fig. 5B). These results indicate that priming with the coexpression vector m8Δ-Env/hCD40Lm markedly enhanced the avidity of the Env antibodies produced, compared to the Env expression vector alone; co-immunization with m8Δ-Env and m8Δ-p7.5hCD40Lm (group G) did not enhance avidity.

Finally, the titer of neutralizing antibodies was determined using pseudotyped viruses coated with gp160 of JR-CSF, tier 1 SF162, and several tier 2 viruses belonging to clade B and C using TZM-bl reporter cells [35], [43]. All m8Δ prime/SeV boost regimens except the control groups K and L elicited neutralizing antibodies against SF162: the mean titers (ID_50_) of the sera prepared from mice primed with m8Δ-Env/hCD40Lm, m8Δ-Env+m8Δ-hCD40Lm, and m8Δ-Env (group I, G and J) were 954, 287, and 205, respectively. The m8Δ-Env/hCD40Lm-primed group I exhibited a significant enhancement of neutralizing antibody titer against SF162 (Fig. 6). Co-immunization with m8Δ-Env and m8Δ-p7.5hCD40Lm (group G) did not show significantly enhanced nAbs to SF162. Sera from untreated mice contained low levels of a non-specific inhibitor to SF162 (titer approximately 100), but the level is insignificant. However, these regimens, irrespective of the inclusion of hCD40Lm, were not effective in eliciting nAbs against tier 2 viruses, including JR-CSF. Immunization twice with the plasmid expressing gp160 followed by boosting with SeV-env did not elicit detectable anti-Env antibody (see Fig. 4), indicating the importance of priming with m8Δ-Env. Priming and boosting with the same m8Δ-Env did not elicit significant titers of nAb (data not shown). To further confirm that the antibodies but not other factors were responsible for the neutralizing activity of the sera, we purified antibodies using protein A Sepharose. All the purified antibodies showed IgG isotypes. We performed neutralizing antibody measurement using TZM-bl reporter cells as described above. The neutralization capacities of purified immunoglobilins showed high identicalness with the neutralizing titer of original sera (R^2^ = 0.777) (Fig. 6B) confirming that neutralizing antibodies were elicited using our m8Δ prime/SeV boost regimens.

An immunological correlate analysis was conducted using Spearman rank correlates to evaluate the relationship between the SF162 neutralization capacity and the avidity of JR-CSF Env binding antibodies; a significant positive correlation was present (R = 0.686, P<0.0001; Fig. 7A). A weaker, but still significant, positive correlation between the nAb titers and the amount of the Env-binding antibodies was identified (R = 0.501, P = 0.0013; Fig. 7B). Moreover, the amounts of Env-binding antibodies showed a positive correlation with the antibody avidity (R = 0.555, P = 0.000377; Fig. 7C). These results verify that better immunized mice produced not only greater amounts of antibody, but also higher affinity antibodies with enhanced neutralizing capacity.

Secretion of anti-Env IgG and IgA into vaginal fluid was examined. Because of the limited amounts of mouse vaginal fluid, fluid from 5–6 mice was mixed for these assays. The amounts of total IgG in the fluid were relatively constant. Fluid prepared from the DNA prime/SeV-env boost group contained 277 ng/ml IgG, the m8Δ-Env prime/SeV-env boost group contained 270 ng/ml, the m8Δ-Env/hCD40Lm prime/SeV-env boost group contained 252 ng/ml, and the m8Δ-Env + m8Δ-hCD40Lm prime/SeV-env group contained 242 ng/ml. Similar levels of anti-gp160 IgG were detected in all the m8Δ primed groups, but no gp160-specific IgG was detected in the DNA primed group (Fig. 8). Anti-Env IgA was not detected in significant amounts in any immunization group (data not shown).

## Discussion

Although researchers have proposed either antibodies or T cells as the most effective means to elicit protective immunity, a central theme of HIV-1 vaccine design now is to elicit coordinated antiviral CD8^+^ cytotoxic T lymphocytes (CTL) to control HIV-1 infection and CD4^+^ T cells that help induce and maintain CD8^+^ and B cell responses [44]–[46]. In this study, we found that a novel immunization schedule including a HIV-1 Env expressing m8Δ prime/SeV boost regimen is able to elicit both Env-specific CD8^+^ T cells and antibodies. These results sharply contrast the results of conventional DNA prime/virus vector boost regimens that have elicited CD8^+^ T cell responses with poor antibody response, which is consistent with the previous report [47]. Moreover, this regime elicited anti-Env IgG secreted into the vaginal fluid. Thus, the m8Δ prime/SeV boost regimen should provide a basis for an improved immunization protocol. Although nAbs against tier 2 viruses were absent, recent reports have suggested that non-neutralizing Env binding antibodies play a role in preventing infection by HIV-1/SIV [9], [48]. Thus, the humoral immunity elicited by the m8Δ prime/SeV boost regimen may also have an improved efficacy against HIV-1 infection. It is an important future theme to elicit broad nAbs against tier 2 viruses on the basis of this regimen.

In m8Δ prime/SeV boost regimen only priming with m8Δ-Env did not elicit any anti-Env antibody before rSeV boost (data not shown), indicating the necessity of rSeV booster. Intranasal Sendai virus was well tolerated and had good immunogenicity [19], making it a good candidate for HIV vaccine. Indeed, involvement of rSeV in prime-boost-boost HIV vaccine strategies had been reported to elicit persistent humoral response in BALB/c mice and rhesus macaques [49] as well as in pre-clinical trials [50]. However, no elicitation of neutralizing antibody has been reported. Our results that m8Δ prime/SeV boost in combination with hCD40Lm adjuvant regimen efficiently elicited neutralizing antibody suggests that this regimen may be a better vaccine strategy.

Recent advances in immunology have shown that several types of molecules may be used as novel adjuvants to enhance the immunogenicity of vaccines. In addition to alum and MF59, other adjuvants including cytokines, chemokines, toll-like receptor ligands and some co-stimulatory molecules may have potential for clinical use [51]–[54]. Among these molecules, CD40L is one of the most potent stimuli for DCs, which activate CTL and B cells [15], [25]–[28]. We showed that hCD40Lm expressing m8Δ in conjunction with Env expressing vaccinia virus enhanced production of HIV-1 specific CTL, but not antibodies. In contrast, the coexpression m8Δ-Env/hCD40Lm vector did not increase the induction of Env-specific CD8^+^ T cells compared to m8Δ-Env alone, but did elicit more anti-Env antibodies with higher avidity and neutralizing capacity against tier 1 SF162. The high avidity antibodies elicited by m8Δ-Env/hCD40Lm may constitute nAb. Indeed, we found a positive correlation between the avidity of Env-binding antibodies and neutralizing activity against HIV-1 SF162 (Fig. 7). The enhancement of antiviral immunity by hCD40Lm in this mouse model also suggests that human CD40Lm is functional in mouse, encouraging us to use it as an AIDS vaccine adjuvant. We have to admit that our novel vaccine regimen still has room to be improved. The species variation between human and mouse CD40Lms should be considered when noting that the enhancement of humoral immunity to be less impressive. Using homogenous CD40Lm or further inclusion of other adjuvant is encouraged to magnitude antibody response of our novel vaccine regimen. Although the reason for the divergent effects of hCD40Lm expressed from different constructs on the production of viral specific immunity is unclear, Given that co-immunization with the two vectors may result in gp160 and CD40Lm being expressed on the same cell or on different cells, whereas immunization with the coexpression vector m8Δ-RCSFenv/hCD40Lm results in gp160 and CD40Lm being expressed on the same cells. Simultaneous expression of gp160 and CD40Lm may preferentially stimulate B cells through a direct interaction with the vector-infected cells because only B cells express both CD40 and a B cell receptor to gp160. The expression of CD40Lm alone may promote the maturation of DCs that were sensitized by gp160 and lead to the activation of cellular immunity. Alternatively, the expression levels of Env and hCD40Lm in cells that have been infected with both m8Δ-Env and m8Δ-hCD40Lm may be different from levels in cells infected with m8Δ-Env/hCD40Lm, leading to different immune responses. Other hypothesis to explain these phenomena cannot be excluded at this point.

The detection of anti-Env IgG in vaginal fluid indicates that our vaccine regimen can also elicit mucosal immunity. However, in contrast to the enhancing effect of hCD40Lm on production of anti Env antibodies in sera, we didn't find that inclusion of hCD40Lm promote levels of anti-gp160 IgG in vaginal fluid as seen in Fig. 8. The reason for this difference is currently under elucidation. Further investigation of mechanism responsible for this divergent effect may be profitable to improve our vaccine regimen to achieve more potent mucosal immunity against HIV.

Recently, monoclonal antibodies (mAbs) that broadly neutralize most HIV-1 strains have been isolated from chronically infected subjects, and analyses of the epitopes recognized by these antibodies may direct the way to devise antigens that elicit broad nAbs [55]–[57]. These studies suggest the possibility of a vaccine that elicits the production of broadly neutralizing antibodies that could prevent HIV-1 infection. However, detailed analyses of broadly neutralizing mAbs have indicated a requirement for extensive affinity maturation of the cognate immunoglobulin genes [57]. The observation that broad neutralizing Abs are generated after a long incubation period in HIV-1-infected individuals also supports this idea and, furthermore, suggests the importance of repetitive antigenic stimulation [58], [59]. Therefore, in addition to devising specific antigens, the development of methods to promote the affinity maturation of antibodies and maturation of B cells should be equally important. This study has demonstrated that CD40Lm, which can activate class switching of immunoglobulin genes and maturation of dendritic cells [22], [23], is suitable for eliciting more potent nAbs. In addition, replication-competent m8Δ that repeatedly presents native antigens in vivo may be important for effective immunization.

In conclusion, this study showed that a novel vaccine regimen that includes the expression of hCD40Lm in the context of LC16m8Δ priming and Sendai virus vector boosting was able to elicit both HIV-1 Env-specific cellular and humoral immunities. Thus, such a regimen may provide a platform for HIV-1 vaccine development, as well as other infectious pathogens.
